# Does the Removal of Textbook Reading from Emergency Medicine Resident Education Negatively Affect In-Service Scores?

**DOI:** 10.5811/westjem.2019.11.44639

**Published:** 2020-02-25

**Authors:** Christine Ju, Joseph Bove, Steven Hochman

**Affiliations:** Saint Joseph’s University Medical Center, Department of Emergency Medicine, Paterson, New Jersey

## Abstract

**Introduction:**

In-service exam scores are used by residency programs as a marker for progress and success on board exams. Conference curriculum helps residents prepare for these exams. At our institution, due to resident feedback a change in curriculum was initiated. Our objective was to determine whether assigned Evidence-Based Medicine (EBM) articles and Rosh Review questions were non-inferior to Tintinalli textbook readings. We further hypothesized that the non-textbook assigned curriculum would lead to higher resident satisfaction, greater utilization, and a preference over the old curriculum.

**Methods:**

We collected scores from both the allopathic In-training Examination (ITE) and osteopathic Emergency Medicine Residency In-service Exam (RISE) scores taken by our program’s residents from both the 2015–2016 and 2016–2017 residency years. We compared scores pre-curriculum change (pre-CC) to scores post-curriculum change (post-CC). A five-question survey was sent to the residents regarding their satisfaction, preference, and utilization of the two curricula.

**Results:**

Resident scores post-CC were shown to be non-inferior to their scores pre-CC for both exams. There was also no significant difference when we compared scores from each class post-CC to their respective class year pre-CC for both exams. Our survey showed significantly more satisfaction, utilization, and preference for this new curriculum among residents.

**Conclusion:**

We found question-based learning and Evidence-Based Medicine articles non-inferior to textbook readings. This study provides evidence to support a move away from textbook readings without sacrificing scores on examinations.

## INTRODUCTION

Each year residents across the country take in-service examinations as a part of their training and preparation for board certification examinations taken at the end of their residency. Specifically, emergency medicine (EM) allopathic residents take the In-training Examination (ITE) and osteopathic residents take a similar test, the EM Residency In-service Examination (RISE). These examinations are used by programs to determine the progress of their residents. Strong correlations exist between these training exam scores and scores on the allopathic Written Qualifying Examination and the osteopathic EM Written (Part I) Exam.^1^,^2^ A plethora of resources are available for the preparation for these examinations including study guides, review books, and online question banks.

Due to resident feedback in the 2015–2016 program year, faculty from the EM residency program at St. Joseph’s University Medical Center in Paterson, New Jersey, met with the incoming academic chiefs to discuss ways to improve the core curriculum. Through an open forum discussion, it became clear that residents were not enjoying the current textbook reading and many times admitted to skipping the assigned reading. It was known that residencies in the surrounding area were using alternative means of learning including *Evidence-Based Medicine (EBM)* articles. Therefore, we made the decision to move away from assigned chapter readings in *Tintinalli’s Emergency Medicine: A Comprehensive Study Guide*.^3^ Instead, the curriculum was changed to *EBM* articles and assigned Rosh Review questions.^4^,^5^

The *EBM* articles served as short, evidence-based reviews of broader concepts to complement the question-based learning from Rosh Review questions. Although cost was not a factor in the decision to change the curriculum, residents in the program had free online access to *Tintinalli’s* through the hospital library. While there was no additional cost to provide access to *EBM* articles, the program paid $3,696 to provide Rosh Review for 24 residents in the 2016–2017 academic year. We believed that the in-service scores would be at least as good after the change as they were before (non-inferior). Secondly, we hypothesized that resident satisfaction would be higher with the change because we believed residents would enjoy non-textbook sources.

## METHODS

We collected scores from both the allopathic ITE and the osteopathic RISE taken by our program’s residents in the 2015–2016 and 2016–2017 academic years. Names corresponding to each score were removed by the residency director to protect resident confidentiality. We obtained national averages for both examinations during these years to serve as a comparison. We then compared scores pre-curriculum change (pre-CC) to those post-curriculum change (post-CC). The post-CC began July 1, 2016.

The original curriculum included monthly chapter assignments from *Tintinalli’s* with a variable number of chapters assigned each time in an effort to cover the textbook in its entirety throughout the course of residency. Senior residents, who were overseen by an assigned core faculty member, were assigned each month to write a 15-question quiz as well as develop an hour-long lecture based on the assigned readings. Although the quiz and lecture were administered to the residents during the last conference of the block, compliance was otherwise not formally monitored. The new curriculum was based on monthly subject content.

Rosh Review questions were chosen at random along with *EBM* articles based on the subject to be covered that block. Answers to quizzes found within the *EBM* articles were submitted to the chief residents by email, and the assigned Rosh questions were due at the end of each block and monitored by the assistant program director. Although Rosh Review was available to residents to be used during the 2015–2016 academic year, there were no assigned questions and its use was not monitored.

A five-question survey created by the authors was sent to the residents regarding their satisfaction, preference, and utilization of the two curricula. The six postgraduate year (PGY)1 residents who had not experienced the curriculum prior to the change were not surveyed. Answers were collected electronically and were kept anonymous. Satisfaction with the curriculum was based on a 0–10 scale with 0 being “not satisfied,” 5 being “neutral,” and 10 being “very satisfied.” Use of either curriculum was also scored based on a similar 0–10 scale with 0 being “never utilized,” 5 being “sometimes utilized,” and 10 being “always utilized.” The survey questions are shown in Table 1.

**Population Health Research Capsule**

What do we already know about this issue?

Strong correlations exist between residency training exam scores and scores on the allopathic and osteopathic emergency medicine (EM) board exams.

What was the research question?

Is a non-textbook reading curriculum non-inferior to traditional textbook readings in preparing EM residents for in-service training exams?

What was the major finding of the study?

The new curriculum was non-inferior to the traditional curriculum. Residents were more satisfied with the new curriculum, used it more, and preferred it.

How does this improve population health?

The more effectively we train emergency physicians, the better equipped they will be to care for patients. We must regularly reassess our teaching methods.

The primary outcome of this study was to determine whether the average scores in each residency class from the exams taken post-CC were non-inferior to the exams taken pre-CC. The secondary outcomes were resident satisfaction with the old vs new curriculum, overall utilization of one curriculum compared to the other and, finally, resident preference for one curriculum over the other. This study was approved by the hospital’s institutional review board.

In 2016 the St. Joseph’s University Medical Center EM residency shifted from dual accreditation by the American Osteopathic Association and the Accreditation Committee on Gradual Medical Education (ACGME), to accreditation solely by the ACGME. Therefore, some of our residents took just the osteopathic or allopathic in-service training exams and some of our residents took both. We compared osteopathic and allopathic scores in two separate analyses.

### Data Analysis

We analyzed osteopathic and allopathic scores separately. Two sample t-tests were used to analyze the scores of different residents whereas we used paired t-tests to analyze scores comparing individual residents in different years. We conducted a one sample t-test to compare the residency’s mean scores to the national mean values. P-values of all of our test results were reported. A standard p-value of <0.05 was considered significant. We performed all tests using R data analysis software (R Foundation for Statistical Computing, Auckland, New Zealand).^6^

## RESULTS

### Osteopathic

We evaluated only the 13 residents who took the osteopathic RISE pre-CC and post-CC. As seen in the two osteopathic columns in Table 2, the residents’ individual scores from the year post-CC were compared directly to the scores they received the year pre-CC.

When comparing the PGY-4 scores post-CC to their own respective scores obtained during their third year pre-CC, we found no significant difference (p=0.2). There was no significant difference in individual PGY-3 scores when compared to their respective scores in their second year (p=0.23). The same was concluded of the PGY-2 scores compared to the scores they obtained in their first year (p=0.1). Comparison of each class’s scores post-CC was made to the respective class year pre-CC (Table 3).

For example, when comparing PGY-3 resident scores in the 2016–2017 year post-CC to the PGY-3 resident scores in the 2015–2016 year pre-CC, we found no difference (p =0.54). The same comparison made for the PGY-2 residents yielded no difference as well (p = 0.89). We compared the average score obtained by all of the residents post-CC to the average score rre-CC. Both the post-CC 2016–2017 and pre-CC 2015–2016 residency averages were compared to the national averages in these years as well (Table 4).

Compared to the average osteopathic resident score pre-CC (209.2), the average resident score post-CC (218.3) was significantly higher (p = 0.009). The national average pre-CC in the 2015–2016 year was 200.7. The national average post-CC was 204.9. Our residency average was greater than the national average both pre-CC (p = 0.016) and post-CC (p<0.001). Although we scored higher than the national average both years, the largest increase above the national average occurred in the post-CC time period.

### Allopathic

A total of 11 allopathic residents took the ITE in both the pre-CC 2015–2016 and the post-CC 2016–2017 examination years. Of those 11, five residents were in their third year during the post-CC 2016–2017 residency year and six were in their second year. We directly compared the individual scores from the post-CC examination year to the scores the residents received during the pre-CC examination year the same way we did with the osteopathic scores (Table 2). When comparing the third-year scores post-CC to their own scores during their second year pre-CC, we found no significant difference (p = 0.09). However, when comparing second-year scores post-CC to their respective scores as first year’s pre-CC, we found that they had performed better (p<0.012).

Comparisons of class-year scores post-CC were made to the same residency level in the pre-CC time period in the same manner as was done with the osteopathic residents (Table 3). When comparing PGY-2 scores post-CC to a different group of PGY-2 scores in the pre-CC 2015–2016 year, we found no difference (p=0.85). The same comparison was made for the PGY-1residents and yielded no difference as well (p=0.46).

We compared the average scores obtained by the residents from the allopathic exam in the post-CC 2016–2017 examination year to the average score obtained pre-CC the year prior, and both post-CC and pre-CC residency averages were compared to the national averages in these years (Table 4). Compared to the average resident score pre-CC, 71.7, the average resident score post-CC, 74.8, showed a positive trend but no significant difference (p=0.15). Meanwhile, the national average held fairly constant during this time period with the pre-CC national average being 75.5 and the post-CC national average being 74.6.

### Satisfaction, Utilization, Preference

A total of 15/18 residents (83.3%) responded to the survey questions. The figure shows the survey results regarding the satisfaction and utilization of the *Tintinalli* curriculum versus the Rosh/*EBM* curriculum.

Overall, residents were more satisfied with the new curriculum compared to the prior curriculum (p = 0.0006). The average satisfaction score with the *Tintinalli* readings was 4.13 compared to 7.12 with Rosh Review and EBM in the new curriculum. Residents used the new curriculum more than the former curriculum (p = 0.002). The average utilization score for the old curriculum was 5.13 compared to 7.6 with the new curriculum. Based on the survey results, residents also preferred the new curriculum, with 80% preferring the new curriculum to the old curriculum.

## DISCUSSION

A correlation has been established between scores achieved by residents on their in-service exams during residency training and their scores on board certification exams.^1^,^2^ The correlation between in-training exam scores and performance on board examinations has been well-documented in a number of different specialties including EM. Levy et al looked specifically at the correlation between scores on the RISE and on the osteopathic Emergency Medicine Written (Part I) Examination.^1^ Using scores from over 400 residents over a four-year period, they were able to establish that the rate of passing on the board exam increased with higher scores on the in-service exam. Therefore, it is paramount that programs train their residents to do well on in-service exams.

Preparation for these exams is an integral part of the educational curriculum for residency programs, but there is no consensus on the optimal strategy. Educational curricula differ vastly between residency programs, and we believe most have some textbook readings to help build core knowledge. Our program moved away from textbook readings in the 2016–2017 residency year with the hypothesis that residents would likely be more satisfied and training scores would not suffer. Many theories have been developed with respect to medical education, and some are specific to adult learners. Most influential and well known are the principles of Malcolm Knowles and his theory of andragogy.^7^ Although it was not a reason for the change in curriculum, it can be argued that moving away from assigned textbook readings to the new curriculum allowed the residents to become more autonomous and self-directed learners. Having Rosh questions and *EBM* articles with content based on cases as well as relatable examples honed in on the residents’ prior clinical experiences, allowing for contextual learning.

Our study demonstrates a significant increase in our average osteopathic scores from pre-CC to post-CC and, comparatively, our residents improved their scores more than the national osteopathic average. Scores rose from 209.2 to 218.3 while the national average went from 200.7 to 204.9. It would be expected that a resident would improve his or her score from one year of residency to the next. However, we could find no data in the literature quantifying the expected improvement in scores in the absence of any change in curriculum. Our findings show a significant increase in scores post-CC, but it remains unknown whether it is more than expected from an additional year of residency training. After analysis of the allopathic exam scores, results mainly showed no significant statistical difference in most comparisons. The only comparison that achieved statistical significance was the comparison of the PGY-2 class scores post-CC to their respective scores as first years’ pre-CC. This finding does not undermine our conclusion of non-inferiority of the change in curriculum.

Overall, our study findings suggest a non-inferiority component to the scores obtained without textbook readings to those obtained with textbook readings. This demonstrates that by removing dedicated textbook readings the scores held constant. Although this may not seem of great value, this observation has many implications. For one, the survey demonstrated that residents were more satisfied with the curriculum change. Therefore, satisfaction improved without ultimately lowering scores and failing to prepare residents for the board exam. Our study is in line with findings from Easton and Bernard who found that residents prefer question-based learning over text-based learning when preparing for the boards.^8^ Removing textbook reading and adding an online question bank such as Rosh Review seemed to be well liked and thus was used more often, as shown in the survey.

There has been a recent trend with question-based preparation gaining popularity over textbook chapter readings. This can be explained by a number of reasons. EM residents may prefer the practicality and portability of using question-bank learning along with being able to familiarize themselves with the format and time constraints associated with the in-service and board certification exams. Our study found a similar preference in test preparation. When looking at the satisfaction of *Tintinalli’s* chapter readings vs the Rosh Review with *EBM* curriculum, residents were more satisfied with the latter. This led to residents using the new curriculum more and ultimately preferring it to the old curriculum.

## LIMITATIONS

This study was limited to the in-service scores of a single residency program. Furthermore, not all residents took the training exam both years; thus, there were a limited number of residents who could be studied for the purposes of this research. Therefore, it is unclear whether the data obtained can be applied widely across all residencies or across specialties. This study did not control for the fact that Rosh Review questions were available for residents in both of the years studied. Additionally, the surveys were subject to recall bias as there was no objective measure of compliance.

Another limitation is that the new curriculum demonstrated no improvement over the old curriculum and, therefore, calls into question the necessity of either curriculum. For instance, residents informally admitted to inconsistently reading the assigned textbook reading in the old curriculum and with the addition of the new curriculum performed the same. However, we believe there to be value in the new curriculum because some residents did partake in the textbook reading of the pre-CC and now used the post-CC more and were more satisfied. A final limitation is that the study looked specifically at in-service scores but did not look at clinical outcome measures.

## CONCLUSION

The new curriculum without dedicated textbook readings demonstrated to be non-inferior to the curriculum containing textbook readings. Residents were significantly more satisfied, used it more, and largely preferred it over the prior curriculum.

## Figures and Tables

**Figure:**
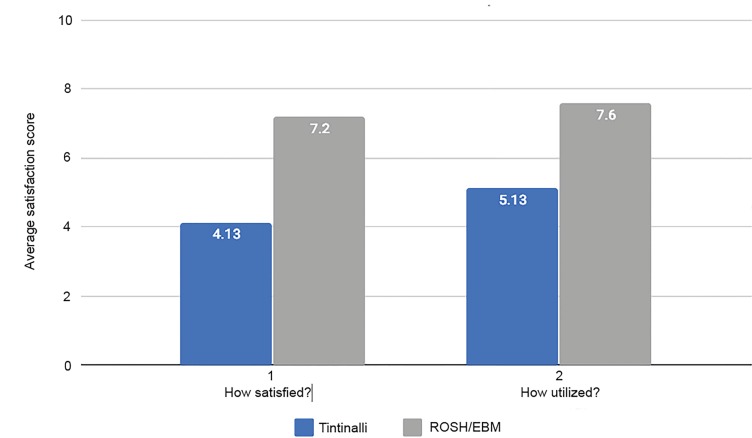
Satisfaction and utilization of the Tintinalli curriculum versus Rosh Review and Evidence-Based Medicine (EBM) curriculum based on survey results.

**Table 1:** Survey questions.

How satisfied were you with the Tintinalli readings assigned as part of the 2015–2016 educational curriculum?
How satisfied are you with the current Rosh/Evidence-Based Medicine (EBM) curriculum?
How often did you utilize Tintinalli during the course of the 2015–2016 year?
How often did you utilize Rosh/EBM during the course of the 2016–2017 year?
If you had to choose between the two, would you prefer to have assigned Tintinalli readings or Rosh/EBM?

**Table 2:** Individual scores for both osteopathic and allopathic residents who participated in the in-service examinations during the 2015–2016 examination year (Pre-CC) and the 2016–2017 examination year (Post-CC).

	Osteopathic in-service score as 3rd year (pre-CC)	Osteopathic in-service score as 4th year (post-CC)	Allopathic in-service score as 3rd year	Allopathic in-service score as 4th year
Resident A	217	224		
Resident B	215	226		
Resident C	211	223		
Resident D	218	226		
Resident E	222	207		
Resident F	212	229		

	Osteopathic in-service score as 2nd year (pre-CC)	Osteopathic in-service score as 3rd year (post-CC)	Allopathic in-service score as 2nd year (pre-CC)	Allopathic in-service score as 3rd year (post-CC)

Resident G	210	215		
Resident H	199	218	79	83
Resident I			75	69
Resident J	204	215	70	81
Resident K	226	221	78	71
Resident L			75	70

	Osteopathic in-service score as 1st year (pre-CC)	Osteopathic in-service score as 2nd year (post-CC)	Allopathic in-service score as 1st year (pre-CC)	Allopathic in-service score as 2nd year (post-CC)

Resident M			75	83
Resident N	202	223	75	79
Resident O	188	193	63	68
Resident P			65	75
Resident Q	196	218	75	75
Resident R			59	69

*Pre-CC*, pre-curriculum change; *Post-CC*, post-curriculum change.

**Table 3:** Class scores for osteopathic and allopathic residents who took the in-service examinations during the 2015–2016 exam year (pre-curriculum change) and the 2016–2017 exam year (post-curriculum change).

	Osteopathic in-service score as 3rd year (pre-CC)	Osteopathic in-service score as 3rd year (post-CC)	Allopathic in-service score as 3rd year (pre-CC)	Allopathic in-service score as 3rd year (post-CC)
Resident A, G	217	215		
Resident B, H	215	218		83
Resident C, I	211			69
Resident D, J	218	215		81
Resident E, K	222	221		71
Resident F, L	212			70

	Osteopathic in-service score as 2nd year (pre-CC)	Osteopathic in-service score as 2nd year (post-CC)	Allopathic in-service score as 2nd year (pre-CC)	Allopathic in-service score as 2nd year (post-CC)

Resident G, M	210			83
Resident H, N	199	223	79	79
Resident I, O		193	75	68
Resident J, P	204		70	75
Resident K, Q	226	218	78	75
Resident L, R			75	69

	Osteopathic in-service score as 1st year (pre-CC)	Osteopathic in-service score as 1st year (post-CC)	Allopathic in-service score as 1st year (pre-CC)	Allopathic in-service score as 1st year (post-CC)

Resident M,S			75	65
Resident N, T			75	57
Resident O, U			63	76
Resident P, V			65	67
Resident Q, W			75	63
Resident R, X			59	66

*Pre-CC*, pre-curriculum change; *Post-CC*, post-curriculum change.

**Table 4:** Average residency scores for both osteopathic and allopathic in-service exams in the pre-curriculum change 2015–2016 and post-curiculum change 2016–2017 years. National averages on both osteopathic and allopathic in-service exams in those years.

	2015–2016 average resident score pre-CC	2015–2016 national average pre-CC	2016–2017 average resident score post-CC	2016–2017 national average post-CC
Osteopathic Score	209.23	200.7	218.31	204.9
Allopathic Score	71.73	75.5	74.8	74.6

*Pre-CC*, pre-curriculum change; *Post-CC*, post-curriculum change.
